# *Campylobacter jejuni* induces autoimmune peripheral neuropathy via Sialoadhesin and Interleukin-4 axes

**DOI:** 10.1080/19490976.2022.2064706

**Published:** 2022-04-20

**Authors:** Ankit Malik, Jean M. Brudvig, Barbie J. Gadsden, Alexander D. Ethridge, Linda S. Mansfield

**Affiliations:** a Department of Large Animal Clinical Sciences, Michigan State University, East Lansing, MI, USA; b Department of Microbiology and Molecular Genetics, Michigan State University, East Lansing, MI, USA; c Comparative Medicine and Integrative Biology Program, Michigan State University, East Lansing, MI USA

**Keywords:** *Campylobacter jejuni*, autoimmunity, neuropathy, Guillain Barré Syndrome (GBS)

## Abstract

*Campylobacter jejuni* is a leading cause of gastroenteritis that has been causally linked with development of the autoimmune peripheral neuropathy Guillain Barré Syndrome (GBS). Previously, we showed that *C. jejuni* isolates from human enteritis patients induced Type1/17-cytokine dependent colitis in interleukin-10 (IL-10)^−/−^ mice, while isolates from GBS patients colonized these mice without colitis but instead induced autoantibodies that cross-reacted with the sialylated oligosaccharide motifs on the LOS of GBS-associated *C. jejuni* and the peripheral nerve gangliosides. We show here that infection of IL-10^−/−^ mice with the GBS but not the colitis isolate led to sciatic nerve inflammation and abnormal gait and hind limb movements, with character and timing consistent with this syndrome in humans. Autoantibody responses and associated nerve histologic changes were dependent on IL-4 production by CD4 T cells. We further show that Siglec-1 served as a central antigen presenting cell receptor mediating the uptake of the GBS isolates via interaction with the sialylated oligosaccharide motifs found specifically on the LOS of GBS-associated *C. jejuni*, and the ensuing T cell differentiation and autoantibody elicitation. Sialylated oligosaccharide motifs on the LOS of GBS-associated *C. jejuni* therefore acted as both the Siglec-1-ligand for phagocytosis, as well as the epitope for autoimmunity. Overall, we present a mouse model of an autoimmune disease induced directly by a bacterium that is dependent upon Siglec-1 and IL-4. We also demonstrate the negative regulatory role of IL-10 in *C. jejuni* induced autoimmunity and provide IL-4 and Siglec-1 blockade as potential therapeutic interventions against GBS.

## Introduction

*Campylobacter jejuni* is a gram-negative foodborne bacterium that affects 1.4 million individuals annually in the United States and is a leading cause of gastroenteritis worldwide.^1^ *Campylobacter jejuni* is ubiquitous in the gastrointestinal (GI) tracts of chickens and food animals^2,3^ and ingestion of contaminated meat or milk results in inflammatory diarrhea of the colon that can be hemorrhagic. The majority of healthy adults with campylobacteriosis experience GI disease for 7–10 days followed by resolution, but it has been a cause of mortality in high-risk individuals.^4,5^ Infection or disease due to *C. jejuni* has also been linked to development and flare-ups of other chronic enteric diseases including Irritable Bowel Syndrome and Inflammatory Bowel Disease.^6,7^

Antecedent infection with *C. jejuni* has also been linked to development of autoimmune diseases including Guillain Barré Syndrome (GBS) and Reactive Arthritis.^8,9^ GBS encompasses a spectrum of autoimmune peripheral neuropathies including Acute Inflammatory Demyelinating Polyradiculoneuropathy (AIDP) and Acute Motor Axonal Neuropathy (AMAN). AIDP primarily involves demyelination of motor axons by inflammatory cell infiltration while AMAN involves axon death without marked inflammatory infiltrates.^10^ Early symptoms of GBS appear as tingling or numbness in the extremities that may rapidly progress to ascending paralysis and can result in death.^11,12^ GBS is the most common cause of acute flaccid paralysis worldwide and *C. jejuni* is the most commonly diagnosed antecedent infection of GBS.^13^ Mortality rates are low at 2.8% of 527 GBS patients in one study and 3.9% of 356 GBS patients in another study.^14^ Up to 90% of patients face long-term disability after recovery from the acute stage of the disease and may experience relapses.^12^

*Campylobacter jejuni* infection is primarily linked with the AMAN form of GBS that has been associated with development of autoantibodies targeting gangliosides on peripheral nerves.^9,15^ Gangliosides are sialic acid containing glycolipid moieties in the myelin sheath and in the outer leaflet of the neuronal plasma membranes. Oligosaccharide motifs on the outer surface of *C. jejuni* endotoxin (lipooligosaccharide) isolated from AMAN patients have been shown to mimic the peripheral nerve gangliosides, namely GM1, GD1a, GQ1b and others.^16,17^ Molecular mimicry is thought to play a role in the production of these anti-ganglioside antibodies, which bind to and attack host nerve tissue. Binding of antiganglioside antibodies elicits complement fixation and infiltration of inflammatory cells leading to tissue damage.

We previously developed a model where GBS was induced in non-obese diabetic (NOD) mice following *C. jejuni* infection.^18^ Infected NOD wildtype and NOD IL-10^−/−^ mice produced anti-ganglioside antibodies of the IgG1 isotype directed against GM1, GQ1b, GD1a and a mixture of GM1/GQ1b gangliosides that produced peripheral neuropathy and peripheral nerve damage. Interestingly, we had previously shown that a number of isolates of *C. jejuni* from human enteritis patients induced colitis in IL-10^−/−^ mice whereas isolates from human GBS patients colonized the IL-10^−/−^ mice but did not induce colitis.^19^ We also showed that the *C. jejuni* induced colitis response depended on an upregulated Type 1 (interferon, IFN-γ) and Type17 (IL-17) cytokine response in the colon. In contrast, *C. jejuni* isolates from human GBS patients did not induce colitis instead eliciting blunted Type1/Type17-cytokine responses. Moreover, GBS isolates induced enhanced Type2 cytokine (IL-4) response. These GBS isolates induced Type2, but not Type1/17 antibodies that cross-reacted with peripheral nerve gangliosides GM1 and GD1a.^20^ However, little is known about the histological or phenotypic consequences of elicitation of these autoantibodies, and the factors that control the contrasting T- and B-cell differentiation following *C. jejuni* colitogenic or GBS isolate infection *in vivo*.

Sialoadhesin (Sn, Siglec-1 or CD169) is a type1 transmembrane protein and a member of the Sialic acid binding Ig-like lectin (Siglec) family. Siglec-1 is expressed by metallophilic macrophages and activated dendritic cells (DC) at the site of afferent lymphatics in the spleen and the lymph nodes, and at the base of the crypts in the colon, which are sites of frequent invasion by pathogens.^21,22^ Therefore, anatomical location of Siglec-1-expressing antigen presenting cells such as DCs and macrophages suggests their role as a sentinel for primary contact with pathogens, and apoptotic or cancer cells. Siglec-1 binds to N-acetylneuraminyl alpha 2-3-galactose (α-2-3 Nan-gal) containing glycolipids and glycoproteins, which facilitates uptake of HIV by activated DC and macrophages *in vitro*.^23,24^ Bax et al. have shown that lipooligosaccharide (LOS) of GBS isolates is α-2,3 sialylated, which facilitates binding to Siglec-1.^16^ Further, this α-2,3 sialylation of the LOS promotes Type 2 maturation in a human DC-T cell coculture system.^25^ A role for Siglec-1 in phagocytosis of *C. jejuni* and primary IFN induction after intra-peritoneal infection has also been demonstrated.^26–28^ Yet, the role of Siglec-1 in *C. jejuni* induced diseases has not been addressed. Further, it is well established that IL-10 is a principal anti-inflammatory mediator for many auto-inflammatory diseases including IBD and multiple sclerosis and the animal model experimental autoimmune encephalomyelitis (MS/EAE).^29^ It has also been shown that naive IL-10^−/−^ mice have a higher number of T cells with autoreactive T cell receptors (TCR) in their lymphoid organs.^30^ Therefore, we hypothesized that *C. jejuni*-induced autoimmune response *in vivo* is mediated by IL-4 and Siglec-1 axes, and these responses are amplified in the absence of IL-10. Experiments were conducted in C57BL/6 and C57BL/6 IL-10^−/−^ models to address this hypothesis.

## Results

### *C. jejuni* induced autoimmunity is IL-4 and T helper cell-dependent

**Figure 1.**
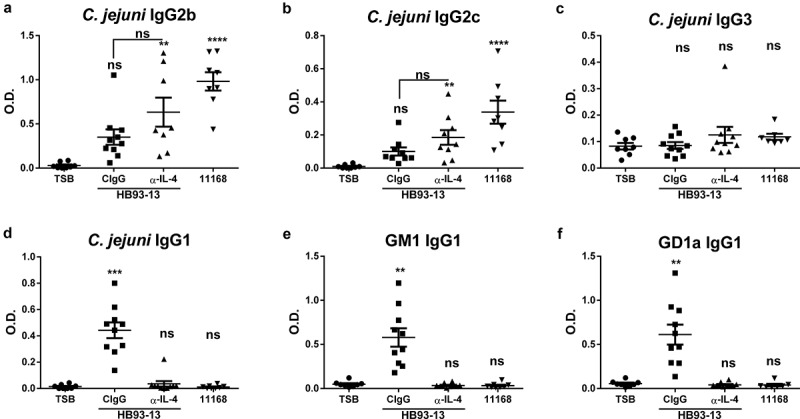
**Depletion of IL-4 during *C. jejuni* infection prevents the elicitation of autoantibodies**. C57BL/6 IL-10^−/−^ mice were orally gavaged with TSB or *C. jejuni* GBS isolate HB93-13 or colitogenic 11168. Sham and HB93-13 infected mice were injected with CIgG or IL-4 neutralizing antibody biweekly for 4 weeks starting at the time of infection. Plasma IgG isotypes reactive to *C. jejuni* antigen (a-d) or peripheral nerve ganglioside autoantigens (e-f) were analyzed by ELISA at day 28. N = 8–10 mice per group. Data are represented as mean ± s.e.m and was analyzed by Kruskal Wallis test and Dunn’s post hoc test.

We have previously shown that *C. jejuni* isolates from human GBS patients colonize the IL-10^−/−^ mice without inducing colitis but do elicit autoantibody production.^20^ These responses were associated with decreased Type1/17 cytokine response, but an enhanced Type 2 cytokine response.^20^ Therefore, we hypothesized that development of autoimmunity in C57BL/6 IL-10^−/−^ mice infected with a *C. jejuni* GBS patient isolate is IL-4 dependent. IL-4 depletion did not significantly increase the Type1/17 dependent IgG2c or IgG2b isotypes (Figure 1(a-b)). Consistent with our previously published results, mice infected with *C. jejuni* GBS isolate HB93-13 and treated with control antibody developed a significant *Campylobacter*-specific Type 2 associated IgG1 response, but not Type1/17 associated IgG2b or IgG2c response at day 28 (Figure 1(d)). Moreover, these Type 2 antibodies also cross-reacted with peripheral nerve gangliosides GM1 and GD1a (Figure 1(e, f)). The extent of both *Campylobacter*-specific and autoreactive IgG1 antibodies were decreased by IL-4 neutralizing antibody administration (Figure 1(d-f)). We have also previously shown that colitogenic isolate 11168 infection followed by IFN-γ and/or IL-17 depletion leads to reciprocal upregulation of *Campylobacter* specific (but not autoreactive) Type2/IgG1 response.^20^ No treatment group developed significant *C. jejuni* specific IgG3 antibodies (Figure 1(c)).

**Figure 2.**
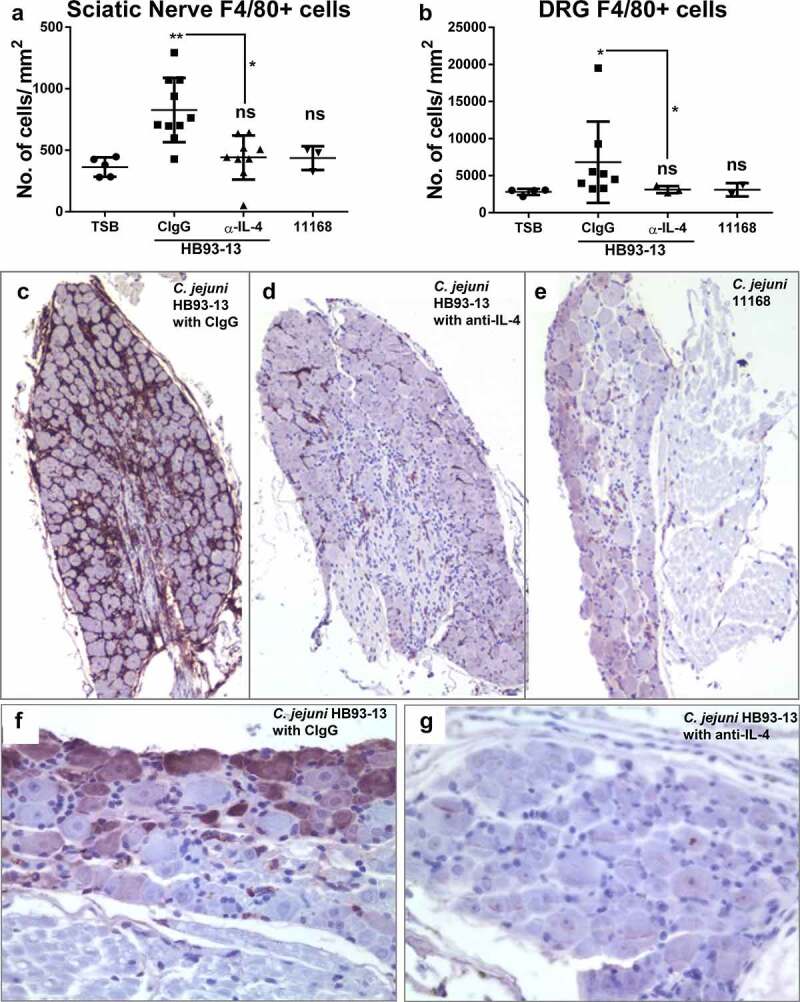
**IL-4 mediates macrophage infiltration in sciatic nerve and DRGs upon infection with the GBS isolates**. C57BL/6 IL-10^−/−^ mice were orally gavaged with TSB or infected with *C. jejuni* HB93-13 (GBS patient) or 11168 (colitis patient) isolates and injected with CIgG or IL-4 neutralizing antibody. Formalin fixed sciatic nerve and dorsal root sections were stained for F4/80 (c-g). Positively staining cells and tissue area were quantified for each group using the ImageJ cell counter and area tools respectively (Panels A and B). N = 8–10 mice per group. Data are represented as mean ± s.e.m and were analyzed by Kruskal Wallis and Dunn’s post hoc tests. Panel C shows a low power image (10X magnification) and Panel F shows a high power image (40X magnification) of a dorsal root ganglion from a mouse representative of mice infected with *C. jejuni* HB93-13 and treated with CIgG. Panel D shows a 10X magnification image and Panel G shows a 40X magnification image of a dorsal root ganglion from a mouse representative of mice infected with *C. jejuni* HB93-13 and treated with anti-IL-4. Panel E shows a 10X magnification image of a dorsal root ganglion from a mouse infected with *C. jejuni* 11168.

We next evaluated peripheral nerves for histologic evidence of disease typically correlated with development of these antiganglioside autoantibodies in mice. In humans, GBS is associated with macrophage infiltration in the sciatic nerves and their roots.^31,32^ Consistent with the human manifestation of the disease, significantly greater numbers of F4/80^+^ macrophages were found infiltrated into the sciatic nerve and the dorsal root in the infected mice when compared to sham-inoculated mice (Figure 2(a-b)). This macrophage infiltration was particularly marked in the dorsal roots (Figures 2(c-f)). Furthermore, this macrophage infiltration was significantly decreased in mice given IL-4 neutralizing antibodies and correlated with decreased autoantibody titer levels in circulation (Figure 2(a-b); Figure 2(d, e, g)).

**Figure 3.**
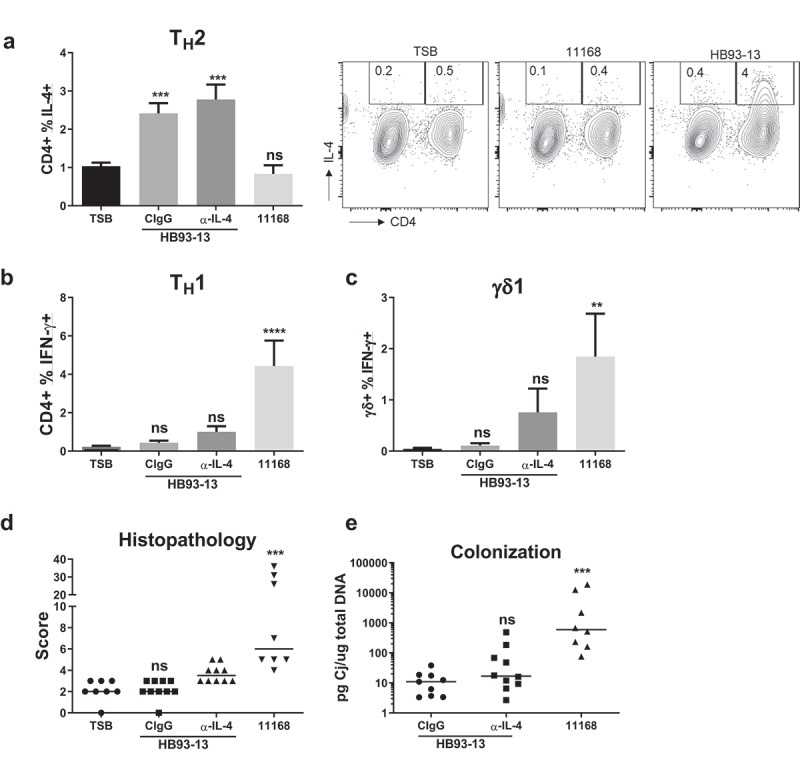
**Role of IL-4 depletion in colon T cell maturation, histologic lesions and *C. jejuni* colonization**. C57BL/6 IL-10^−/−^ mice were orally gavaged with TSB or infected with *C. jejuni* HB93-13 (GBS patient) or 11168 (colitis patient) isolates and injected with CIgG or IL-4 neutralizing antibody. Single cell suspensions of colon leukocytes were prepared at day 28 and analyzed for indicated cell populations by ICCS and flow cytometry (a-c). Formalin fixed ileocecocolic junctions were stained with hematoxylin and eosin and scored for lesions in the colon by our established criteria.^33^ (d). Colonization load was determined by Q-PCR on fecal DNA with *C. jejuni* specific primers (e). Mice infected with the *C. jejuni* colitogenic isolate 11168 had the highest levels of colonization. N = 8–10 mice per group. Data are represented as mean ± s.e.m and were analyzed by Kruskal Wallis test followed by Dunn’s post hoc test. To exclude dead/dying cells and therefore nonspecific antibody-binding cells, leukocytes were gated according to forward and side scatter. The percentages of CD4^+^, CD8^+^, CD4^+^ CD8^+^ and γδT cells subsets were calculated on CD19^−^ CD3^+^ gate.

Previously we have also shown that both innate and adaptive lymphocytes contribute to upregulated production of IFN-γ, IL-17 and IL-22 after colitogenic isolate infection.^20^ In contrast, we found here that after GBS isolate infection, upregulation of IL-4 was mainly restricted to the CD4^+^ T cells at day 28 (Figure 3(a-c)). Therefore, *C. jejuni* induced autoimmunity is IL-4 and CD4 T cell-dependent. However, IL-4 depletion did affect Type 1 responses, but did not induce inflammation in the colon or clinical signs of colitis in these mice (Figure 3(d)). We have also previously shown that colitis induction by colitogenic isolates in IL-10^−/−^ mice correlates with an increase in their colonization level.^20^ However, IL-4 depletion did not affect colonization levels of the *C. jejuni* HB93-13 GBS isolate (Figure 3(e)). Therefore, lack of colitis induction after infection with the *C. jejuni* HB93-13 GBS isolate is independent of the Type 2 immune response.

### Siglec-1 is essential for GBS-isolate uptake, and cytokine and autoantibody elicitation

**Figure 4.**
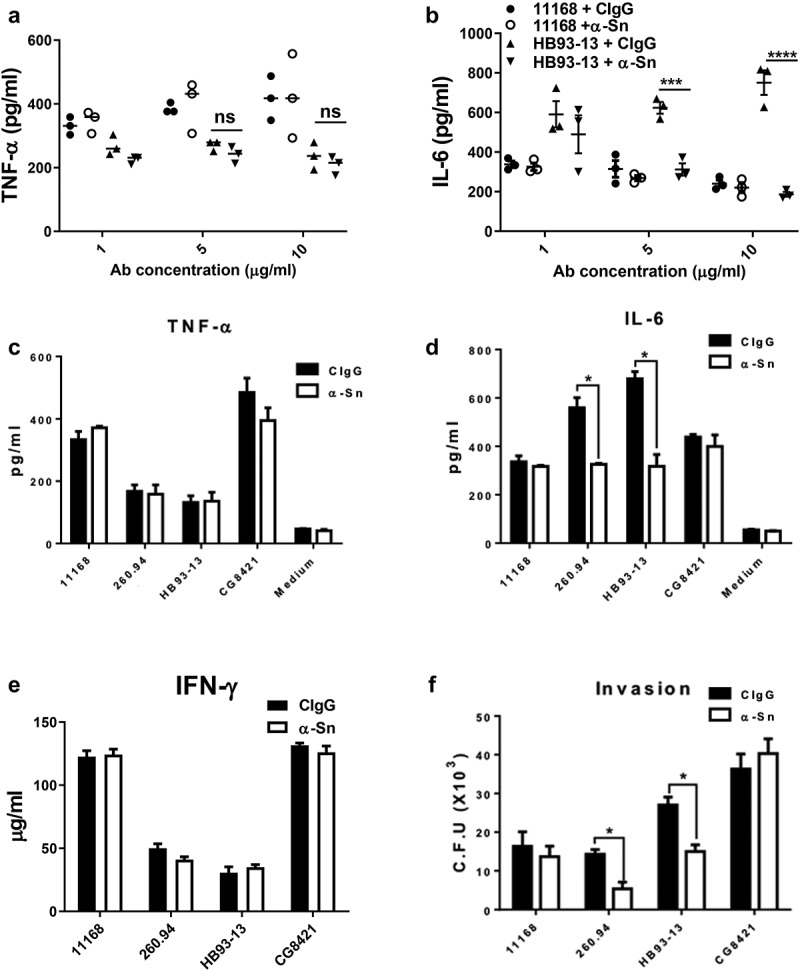
**Siglec-1 mediates the innate immune response specifically for the GBS isolates**. Single cell suspension of whole splenocytes from naïve C57BL/6 mice were challenged with the indicated *C. jejuni* isolates at an MOI of 1 by gentamycin killing assay. Cells were pretreated for 20 minutes by anti-Siglec1 or control antibody (1, 5 or 10 μg/ml for A; 5 μg/ml for B). Gentamycin was added 1 hour after challenge and 72 hours later. Indicated cytokine levels were determined by ELISA in clarified supernatant media (a-e). For invasion assay, cells were lysed 1 hour after washing off gentamycin (f). Gentamycin sensitivity to all the strains was also confirmed. Data represent mean ± s.e.m of three wells, and experiments were repeated at least twice independently. Data were analyzed by two-way ANOVA followed by Bonferroni’s post hoc test.

It has been demonstrated that *C. jejuni* LOS with α-2,3 sialylation is structurally similar to sialylated peripheral nerve gangliosides, which acts as a ligand for Siglec-1.^25^ However, the role of Siglec-1 in modulating adaptive T or B cell responses during infection has not been explored. We used a naïve splenocyte gentamycin killing assay to determine the role of Siglec-1 in uptake and immune stimulation by GBS (HB93-13) and colitogenic (11168) *C. jejuni* isolates. Treatment with anti-Siglec-1 antibody, but not the isotype control antibody, significantly decreased IL-6 elicitation from GBS isolates in a dose-dependent manner but had no effect on TNFα responses (Figure 4(a-b)). Consistent with our previous observations,^20^ GBS isolates HB93-13 and 260.94 induced more IL-6 while the colitogenic isolates 11168 and CG8421 induced more TNF-α and IFN-γ production (Figure 4(c-e)). Also, consistent with the lack of α-2,3 sialylation on the LOS colitogenic isolates 11168 and CG8421, cytokine production during their infection was not affected by Siglec-1 blockade (Figure 4(a-e)). Furthermore, elicitation of Type1 cytokines like TNF-α and IFN-γ from GBS or colitogenic isolates was not affected by Siglec-1 blockade (Figure 4(a, c-e)). Siglec-1 is a cell surface receptor known to be involved in uptake of HIV by macrophages and DC.^23,24^ Therefore, we hypothesized that Siglec-1 blockade will lead to similarly decreased uptake of GBS but not colitogenic isolates. Consistent with this hypothesis, Siglec-1 blockade significantly reduced uptake of GBS but not colitogenic isolates (Figure 4(f)). To corroborate the specificity of the Siglec-1 receptor toward ganglioside-presenting *C. jejuni* isolates, we also included an isolate that lacks all ganglioside mimics, *C. jejuni* CG8421, in the cytokine elicitation and invasion assay. We have previously shown that CG8421 causes a high degree of colitis in IL-10^−/−^ mice that is consistent with its high Type1 and 17 cytokine elicitation characteristic.^20^ Consistent with lack of ganglioside presentation on its surface,^34^ Siglec-1 blockade did not affect cytokine elicitation (Figures 4(c, d-e)) or uptake (Figure 4(f)) of CG8421 in whole and adherent splenocytes, respectively.

**Figure 5.**
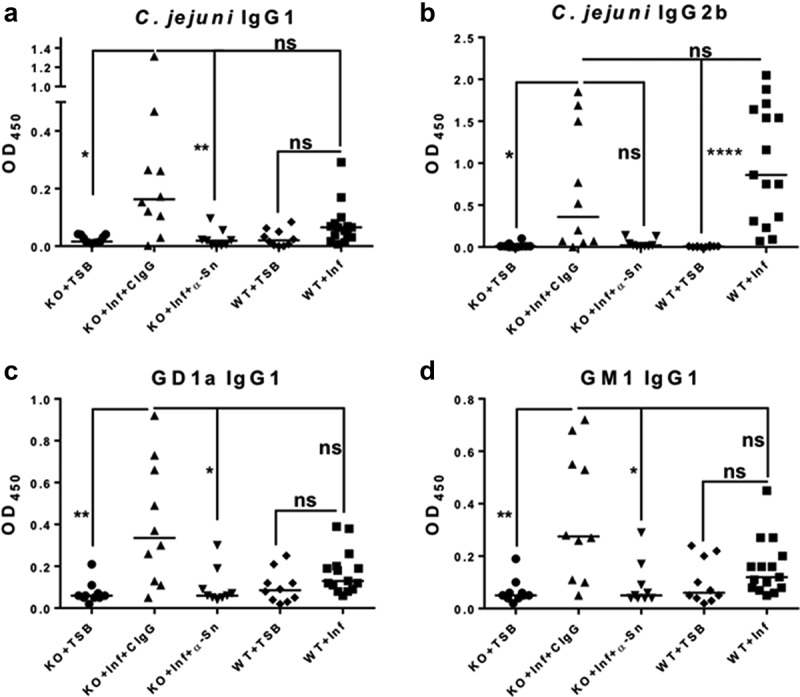
**Siglec-1 mediates *C. jejuni* specific and autoantibody production**. C57BL/6 wildtype (WT) or IL-10^−/−^ knockout (KO) mice were orally gavaged with TSB or GBS patient isolate *C. jejuni* HB93-13 (Inf) and injected with CIgG or anti-Siglec-1 antibody weekly for 6 weeks starting at two days before infection. Plasma IgG isotypes reactive to *C. jejuni* antigen (a) IgG1 and (b) IgG2b or peripheral nerve ganglioside autoantigens (c) GD1a IgG1 and (d) GM1 IgG1 were analyzed by ELISA at the time of necropsy. N = 9–15 mice per group. Data were analyzed by Kruskal Wallis followed by Dunn’s post hoc test. Horizontal bar represents the median.

To address the role of Siglec-1 in *C. jejuni* induced GBS, we administered anti-Siglec-1 antibody for six weeks in the HB93-13 infected IL-10^−/−^ mice. Consistent with the hypothesis, Siglec-1 blockade for 6 weeks during GBS isolate infection significantly decreased Type 2 differentiation in the colon (Figure 5(a)). Consistent with blunted Type2 immune response, *Campylobacter*-specific (Figure 5(a)) and autoreactive anti-GM1 and anti-GD1a IgG1 antibody elicitation (Figures 5(c-d)) was also significantly decreased by Siglec-1 neutralization. However, the effect of Siglec-1 blockade was not Type 2 specific as anti-*C. jejuni* IgG2b level also trended toward a decrease (Figure 5(b)). Therefore, Siglec-1 receptor specifically promotes uptake, T cell maturation and autoantibody induction by the GBS isolates of *C. jejuni*. Finally, consistent with our previous data,^20^ 2/10 mice in the infected + control immunoglobulin treated (CIgG) group that failed to be colonized at the end of the experiment were low/negative for *C. jejuni* specific or autoreactive antibodies. One mouse in the infected + Siglec-1 blocked group failed to be colonized at the end of the experiment.

### IL-10 is a negative regulator of autoantibody production

We have also previously shown that the colitogenic isolate *C. jejuni* 11168 colonizes IL-10^+/+^ mice asymptomatically.^33^ However, the infected IL-10^+/+^ mice exhibit anti-*Campylobacter* antibodies of the same classes (IgG2b, IgG2c and IgG3) as the IL-10^−/−^ mice.^20^ Therefore, we aimed to determine if IL-10 affects autoantibody production during infection with a GBS isolate. To this end, C57BL/6 IL-10^+/+^ mice were either sham inoculated or inoculated with HB93-13 (alongside IL-10^−/−^ mice from the Siglec-1 blocking experiment) and analyzed for *C. jejuni*- and autoreactive antibody elicitation 6 weeks post-infection. Infected IL-10^+/+^ mice developed a significant anti-*Campylobacter* IgG2b response when compared to sham inoculated mice. However, these anti-*Campylobacter* IgG2b levels were not significantly different in the IL-10^−/−^ and IL-10^+/+^ mice (Figure 5(b)). Intriguingly, the Type 2 dependent IgG1 response, both *Campylobacter*-specific and autoreactive with gangliosides GD1a and GM1, was not induced to a significant extent in the IL-10^+/+^ mice (Figure 5(a, c-d)). Therefore, IL-10 functions as a negative regulator of the *C. jejuni* induced Type 2 response and subsequent autoantibody production but does not affect the anti-*C. jejuni* IgG2b response.

### GBS isolate infection leads to abnormal hind limb movements in a subset of IL-10^−/−^ mice

**Figure 6.**
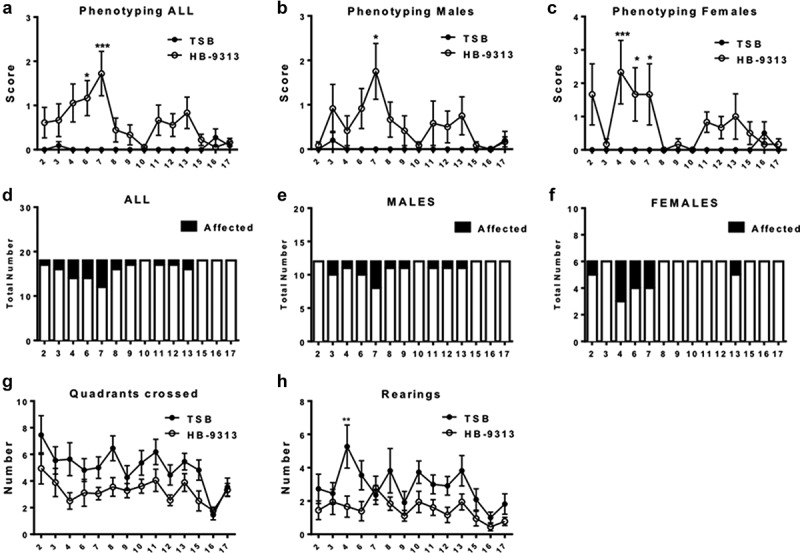
**Long-term phenotyping in GBS isolate infected IL-10^−/−^ mice**. C57BL/6 IL-10^−/−^ mice were orally gavaged with TSB or GBS patient isolate *C. jejuni* HB93-13 and phenotyped weekly for 17 weeks. N = 11–18 mice per group. Error bar represents mean ± s.e.m. Data were analyzed by two way ANOVA followed by Bonferroni’s post hoc test. Panels A to C show the phenotyping scores. Panels D to F show the number of abnormal neurological events shown by the mice. Panel G shows the number of quadrants crossed by mice in the two infection groups. Panel H shows the numbers of rears during open field testing by mice in the two infection groups.

Having shown that IL-10^−/−^ mice infected with GBS isolates develop autoantibodies and have macrophage infiltration in sciatic nerves and its dorsal root ganglia, we evaluated the phenotypic consequences associated with these responses. To this end, sham and GBS isolate HB93-13 infected IL-10^−/−^ mice were subjected to reaching reflex and open field testing weekly, for 17 weeks post-infection, as described in the methods section. The average neurological scores were increased at 4–7 weeks post-infection (Figure 6(a-c)). The incidence of neurologically affected mice varied from 0 to 6 (out of 18) for infected mice, and 0 (out of 11) for sham-inoculated control mice (Figure 6(d-f)). The highest incidence of neurological signs was observed between 4–7 weeks post infection (Figure 6(d-f)). While the infected mice trended toward decrease in the number of quadrants crossed, it did not reach statistical significance (Figure 6(g)). However, the numbers of rears during the open field testing were significantly decreased in infected mice compared to sham inoculated mice at 4 weeks post infection (Figure 6(h)). DigiGait^TM^ analysis on the mice was also attempted, as described previously.^35^ However, these mice gradually stopped cooperating with running on the treadmill at any speed. After the fourth trial (including one trial before infection and 3 weekly trials after infection) only 36% of the infected and 45% of the control mice ran on the treadmill, consistent with a previous finding of recalcitrance of this strain of mice toward treadmill running.^36^ Nevertheless, these data from other neurological phenotyping methods demonstrate that GBS isolate infection leads to certain clinical signs in a subset of the infected mice.

**Figure 7.**
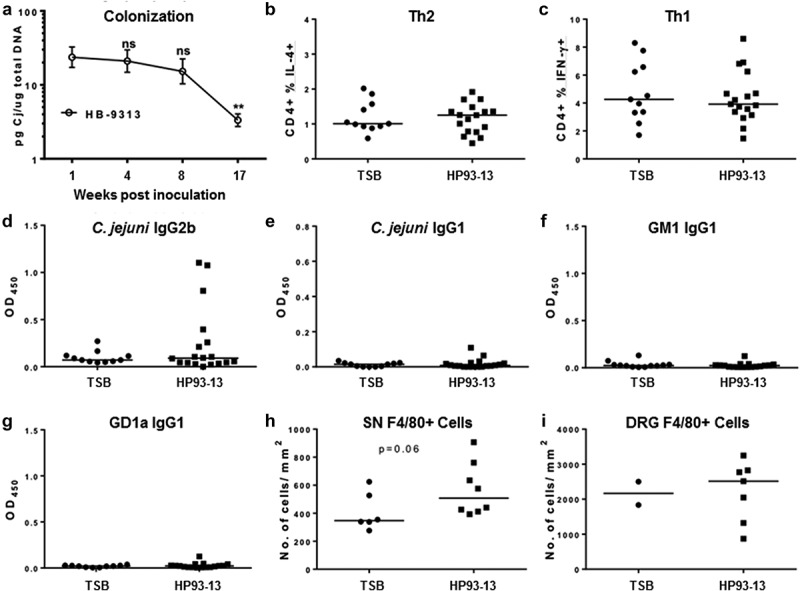
**Long-term colonization, colon T cell maturation and plasma antibody analysis in GBS isolate infected IL-10^−/−^ mice**. C57BL/6 IL-10^−/−^ mice were orally gavaged with TSB or *C. jejuni* HB93-13 and phenotyped weekly for 17 weeks. Colonization load was determined by Q-PCR on fecal DNA with *C. jejuni* specific primers (a). A single cell suspension of colon leukocytes was prepared and analyzed for indicated cell populations by ICCS and flow cytometry.^20^ Plasma IgG isotypes specific to given antigens were quantified by ELISA at the time of necropsy. Formalin fixed sciatic nerve (SN) and dorsal ganglia root sections (DRG) were stained for F4/80. Positively staining cells and tissue area were quantified using the ImageJ cell counter and area tools, respectively, for a subset of mice showing neurological signs. N = 11–18 mice per group. Colonization data were analyzed by one way ANOVA followed by Bonferroni’s post hoc test. Mann-Whitney testing was used for the rest of the analyses.

We have also previously shown that the colonization level of colitogenic isolates increases with time, and that this increase correlates with increasing extent of inflammation in the colon.^33^ However, in this study, colonization with the GBS isolate decreased with time, as the extent of *Campylobacter-*specific DNA in feces decreased significantly at 17 weeks post-infection when compared to 1, 4 or 8 weeks post-infection (Figure 7(a)). This decrease in colonization at 17 weeks post infection correlated with basal levels of Type 2 and Type 1 cells in the colon (Figures 7(b-c)), *Campylobacter* specific IgG2b antibodies (Figure 7(d)) and IgG1 antibodies (Figure 7(e)), and GM1 and GD1a IgG1 autoreactive antibodies (Figure 7(f-g)) in circulation and a decrease in the neurological signs of the disease at the time of necropsy (Figure 6(a-h)). We also determined the histological manifestations of long-term infection in the sciatic nerve and dorsal roots. While no gross differences were observed upon evaluation of the formalin fixed and H&E stained sections, slightly increased numbers of macrophages were found infiltrated into the sciatic nerve, but not the dorsal root, of the mice that had demonstrated neurologic clinical signs (Figures 7(h-i)). Therefore, *C. jejuni* induced peripheral neuropathy in C57BL/6 IL-10^−/−^ mice is transient as seen in many GBS patients.

## Discussion

We show here that the *C. jejuni* induced autoimmune response is dependent upon blunted Type1/17 but enhanced Type2 cytokine production. The presence of autoantibodies in circulation correlates with enhanced macrophage infiltration in the sciatic nerve and its dorsal root ganglia, while the phrenic nerve was largely unaffected (data not shown). These findings are consistent with human clinical reports of the IgG1 isotype as the most commonly associated autoreactive isotype in *C. jejuni* infection. Further, the titer of auto-reactive IgG1 directly correlates with the severity of clinical signs and worsening prognosis in GBS.^37,38^ Macrophage infiltration is also most marked in DRGs, consistent with its weaker nerve blood barrier.^39,40^ This contrasts with our previous findings that *C. jejuni* mediated colitis in specific pathogen free mice is T cell, IFN-γ and IL-17 dependent^20^ and demonstrates how differential T cell maturation by different *C. jejuni* strains leads to different disease outcomes in a susceptible host. Further work is required to better describe the immune infiltrate in the DRGs and draining lymph nodes and to determine how autoantibodies lead to the infiltration of immune cells.

Siglec-1 is highly expressed by circulating myeloid and local lymph node cells^41,42^ and targeted delivery of microbial/tumor antigens through Siglecs has shown promise for inducing effective T cell and induced natural killer cells (iNKT) activation.^43,44^ Recently it has also been shown that Siglec-1 plays an important role in phagocytosis, and in primary IFN and early cytokine production after challenge with sialylated pathogens.^26,27,45^ We also demonstrate a direct role for Siglec-1 in promoting uptake of *C. jejuni* strains from GBS patients but not strains from colitis patients. Consistent with our findings, Heikema et al., have also shown that anti-Siglec-1 treatment decreases heat killed-GBS isolate uptake and IL-6 (but not TNF-α) elicitation from human blood monocyte-derived and LPS primed macrophages *in vitro*.^28^ However, without a Siglec-1 reporter mouse or an independent anti-Siglec-1 antibody clone, it is unclear if this anti-Siglec-1 treatment causes depletion of these cells or blockade of the receptor. We nevertheless extend these findings to demonstrate a critical role for Siglec-1 in colonic T cell maturation and autoantibody elicitation during *C. jejuni* GBS isolate infection. Therefore, sialylated oligosaccharide motifs on the LOS of GBS-associated *C. jejuni* act as both the Siglec-1-ligand for phagocytosis, as well as an epitope for autoimmunity. Further, Siglec-1 blockade may represent a therapeutic option in GBS. Siglec-1 has also been shown to be highly expressed by circulating myeloid and local lymph node cells in multiple sclerosis and other autoinflammatory diseases in humans^41,42^ and plays a critical pro-inflammatory role by binding to T regulatory cells and preventing their expansion, as shown in the corresponding experimental autoimmune encephalomyelitis (EAE) model.^46^ Further, targeting delivery of microbial/tumor antigens through Siglecs has shown promise for inducing strong T cell and iNKT activation.^43,44^ Therefore, Siglec-1 is a unique receptor involved in both phagocytosis and the antigen presenting cell – T cell interaction. While we have demonstrated a direct role for Siglec-1 in sialylated *C. jejuni* uptake, it remains to be determined if it has an independent role in blocking APC-T cell or T cell-B cell interactions. Nevertheless, sialylated oligosaccharide motifs on the LOS of GBS associated *C. jejuni* are unique in the sense that they act as the ligand for phagocytosis by antigen presenting cells, and also as epitopes that lead to autoimmunity.

Histopathologic damage in the sciatic nerve manifested with abnormal gait and hind limb movements in a subset of infected IL-10^−/−^ mice, consistent with this syndrome’s manifestation in humans. The reason for mild rather than severe disease induction in this subset of infected mice with clinical signs is not clear. Nevertheless, the extent of macrophage infiltration was significantly decreased in mice given IL-4 neutralizing antibodies, without induction of colitis. Therefore, IL-10^−/−^ mice serve as models of *C. jejuni* induced colitis, autoantibody elicitation and sub-clinical inflammation in the peripheral nervous system but are an insufficient model for studying severe clinical changes associated with GBS such as paralysis and respiratory insufficiency. Therefore, this model combines the most frequent genetic perturbation underlying inflammatory disorders (IL-10) with the most common causative organism of colitis and GBS (*C. jejuni*) through its natural route of infection (oral). We also demonstrate the negative regulatory role of IL-10 in *C. jejuni* induced autoimmunity and provide IL-4 and Siglec-1 blockade as potential therapeutic interventions against GBS.

## Materials and methods

### Mice, inoculation, and antibodies for in vivo neutralization

All animal experiments conformed to National Institutes of Health guidelines and were approved by the Michigan State University Institutional Animal Care & Use Committee under protocol numbers 04/07- 030-00, 06/09-092-00 and 06/12-107-00. C57BL/6 J IL-10^+/+^ (IL-10^+/+^; wildtype) and BL/6.129P2-*IL-10^tm1Cgn^/*J (IL-10^−/−^) mice were purchased from The Jackson Laboratory. Mice were transported to the University Research Containment Facility and maintained in specific pathogen free conditions until experimental use at 8–12 weeks of age. One week before start of the experiment, mice were housed individually and allowed to acclimate for 1 week. On Day 1 of the experiment, mice were inoculated with tryptone soy broth (TSB, vehicle control) or 10^9^ colony forming units (CFU) of *C. jejuni* in 0.2 mL TSB as described previously.^33^ Several strains of *C. jejuni* were used, three from GBS patients (HB93-13, 260.94 and CG8421) and one from a patient with gastroenteritis (11168) that have been previously characterized for phenotype and immune responses in mouse models.^18,20^ Blocking antibodies or the sham control were given according to group. α-Siglec-1 (3D6.112) and isotype control (RTK2758) were purchased from Biolegend (San Diego, CA) and injected retro-orbitally, 100 μg/mouse, weekly for 6 weeks starting at 2 days before inoculation. α-IL-4 (11B11) was purchased from Bio-X-Cell (West Lebanon, NH) and injected intraperitoneally at 400 μg/mouse biweekly, starting at the day of inoculation. Quantitative-PCR for the quantification of *C. jejuni* in fecal pellets was performed with *C. jejuni*-specific gyrA primers CTTTGCCTGACGCAAGAG and TCGCTTTCTGAACCATCA and iQ SYBR green supermix using Tm of 60C and recommended cycling conditions.^47^

### Neurological phenotype testing

All mice were examined in their home cage daily for clinical signs including evidence of neurological deficits. All mice were phenotyped weekly specifically for neurological signs starting 2 weeks before initiation of the experiment to allow for acclimation to the tests. During the reaching reflex, mice were videotaped while being hung upside down by the tail for 5–10 seconds and scored blinded for abnormal hind leg splay (spread legs out to the side and raised up toward the tail, no longer remaining in line with the forelimbs) and leg flexing (hind limbs pulled toward body or making a ‘fist’ with hind limb paw). For the open-field test, mice were placed in a clear rat cage divided into 4 quadrants and videotaped for two minutes. Blinded videotapes were scored for wide gait stance, foot drag, and knuckling. Mice were considered affected if they were scored positively for one or more of the above features. The number of rears and quadrants crossed in the open field test were quantified separately. All positive features were added to achieve a total score for each mouse on each day of assessment.

### Immunohistochemical scoring of mononuclear cells in sciatic nerve and dorsal root ganglia

At necropsy, the hind limbs were dissected to expose the sciatic nerves and dorsal root ganglia from the spinal cord (L3 to L5) to the tibial nerve according to our standard protocol.^18^ We were able to dissect three dorsal root ganglia from the majority of mice. Nerve tissues were removed and embedded enbloc to allow for assessment of cellular infiltrates that can be segmental. Details of the dissections of sciatic nerves and dorsal root ganglia are published.^18^ Tissue specimens were fixed in 10% formalin buffer pH 7.0 for 24 hours, changed to 60% ethanol and, thereafter, paraffin embedded. Sections were stained for F4/80 as described previously.^20^ For each section at 20X magnification, contiguous fields of view were photographed sequentially using a Nikon Eclipse E600 microscope with a SPOT camera with Windows TM version 4.09 software (RTSlider Diagnostic Instruments, Inc., Sterling Heights, MI) to include the entire area of the sciatic nerve and the dorsal root ganglion. Dissections and photography of images were performed by a board-certified veterinary pathologist (JMB). Positively staining cells were counted using the free hand and cell counter tool of ImageJ tool (N.I.H) by a second trained operator (AM) who was blinded to identity of the samples during counting.

### Preparation of lamina propria (LP) lymphocytes

LP lymphocytes were isolated as previously described.^20^ Briefly, for removal of epithelial cells, the colon was washed and cut into small pieces which were then incubated with calcium- and magnesium-free Hank’s Balanced Salt Solution (HBSS) supplemented with 5% fetal bovine serum (FBS) and 5 mM ethylenediaminetetraacetic acid (EDTA) (Sigma-Aldrich) at 140 rpm at 25°C for 30 min. The tissues were then incubated with RPMI 1640 containing 10% FBS and 0.5 mg/ml collagenase type IV for 1 hour at 37°C with shaking at 150 rpm. The liberated cells were collected by passage through a 70 μm nylon mesh. The isolated cells were pooled together and separated on a 40/80% discontinuous Percoll gradient (GE Bioscience). The cell yield was typically 1–2 × 10^6^ cells per mouse with 90% cell viability, as ascertained by Propidium Iodide staining.

### Flow cytometry

The following monoclonal antibodies (eBiosciences) were used in appropriate combinations: anti-CD3 (clone 145–2C11), anti-CD4 (clone RM 4–5), anti-TCR γδ (clone GL3), anti-CD19 (clone 1D3), anti-CD11b (clone M1/70), anti-Gr1 (clone-RB6-8C5), eFlour 780 anti-CD90 (clone53-2.1) and anti-CD16/CD32 (clone 2.4G2). The cells were preincubated for 20 minutes with anti-CD16/CD32 to block Fc receptors to avoid nonspecific binding. Cells were then washed and labeled with the appropriate mixture of antibodies or isotype matched controls for 30 minutes, centrifuged at 650 *g*, and resuspended in flow cytometry staining buffer (FACS) buffer. To exclude dead/dying cells and therefore nonspecific antibody-binding cells, leukocytes were gated according to forward and side scatter. The percentages of CD4^+^, CD8^+^, CD4^+^ CD8^+^ and γδ T cell subsets were calculated on a CD19^−^ CD3^+^ gate. For intracellular cytokine staining, cells were restimulated for 4 h with 50 ng/ml PMA and 1 µg/ml Ionomycin (Sigma) and Golgi Stop and block (BD biosciences) were added for the last two hours. The cells were fixed and permeabilized using fixation and permeabilization solution (eBiosciences). Staining was performed for IL-4 (clone 11B11) and IFN-γ (clone XMG1.2) antibodies, and the cells were analyzed on a LSRII flow cytometer (BD Biosciences) using FlowJo software (Tree Star).

### Enzyme-linked immunosorbent assay

IFN-γ, IL-6 and TNF-α, were measured in tissue culture supernatants according to the manufacturer’s protocol (Ready-Set-Go ELISA kits, eBiosciences). *C. jejuni* and anti-GM1 and GD1a antibody ELISA was used as described earlier.^20^ Briefly, the protein concentration was adjusted to 1.9 μg/ml. Nunc-Immuno Maxisorp plates were coated with the antigen overnight at 4°C, then blocked overnight at 4°C in blocking buffer (3% BSA in PBS with 0.05% Tween 20). The next day, plates were washed 4 times with wash buffer (PBS with 0.05% Tween 20). Plasma samples diluted in blocking buffer (or blocking buffer alone as negative control) were applied to the plate and incubated overnight. The next day, plates were washed 4 times and incubated with biotinylated anti mouse IgG1, IgG2b, IgG2c, IgG3 or IgM (Jackson Imumunoresearch) at 1:20000 dilution in blocking buffer, incubated for 1 hour at room temperature, washed 4 times with wash buffer and incubated for 1 hour at room temperature with ExtrAvidin Peroxidase reagent (Sigma) diluted 1:2,000 in PBS with 1%BSA and 0.05% Tween 20. Plates were washed four times, developed with TMB (Sigma), stopped with 2 N sulfuric acid and absorbance at 450 nm was read with 562 nm as the reference wavelength. Plasma dilutions were 1:25 for IgG1, IgG2c and IgG3; 1:100 for IgG2b. Only absorbance values more than 3 SD away from mean of negative control were considered positive. GM1 (Sigma) and GD1a (US Biologicals) were used at 2 and 20 µg/ml respectively and handled similarly.

### Splenocyte challenge by gentamicin killing assay

Red blood cell-depleted splenocytes from naive C57BL/6 mice (3 × 10^6^ cells/ml) were plated in antibiotic-free R10 medium and challenged with the indicated *C. jejuni* strains at a multiplicity of infection (MOI) of 1. We used several *C. jejuni* strains including HB93-13, 260.94 and CG8421 from GBS patients and 11168 from a patient with gastroenteritis.^18,20^ One hour after infection, gentamicin (250 μg/ml) was added to all the wells to kill extracellular bacteria. Supernatants were collected after 72 h for cytokine measurement by ELISA. To obtain adherent cells, splenocytes were plated at 1 × 10^7^ cells/ml for 90 min after which the non-adherent cells were washed off. For measuring *C. jejuni* invasion, cells were incubated for 1 h with 250 µg/ml gentamicin, washed in PBS, lysed in 0.1% Triton X-100, and released bacteria were enumerated by limiting serial dilution assay. Sensitivity of all strains to this concentration of gentamicin was also confirmed.

### Statistical analyses

All statistical tests were performed in Prism 6.0 (GraphPad Software) and described in short in the figure legends; *p < 0.05, **p < 0.01, ***p < 0.001, ns not significant. Data for plasma IgG isotypes reactive to *C. jejuni* antigen or peripheral nerve ganglioside autoantigens are represented as mean ± s.e.m and were analyzed by Kruskal Wallis and Dunn’s post hoc analyses. Similarly, F4/80-stained cells in sciatic nerve and dorsal root ganglia, T cell subpopulations, histologic scores in the colon, and *C. jejuni* colonization rates were also analyzed using Kruskal Wallis followed by Dunn’s post hoc test. Single cell suspensions of spleen cell data represent the mean ± s.e.m of three wells repeated at least twice independently and analyzed by two-way ANOVA followed by Bonferroni’s post hoc test. Neurological phenotype data was analyzed by one way ANOVA followed by Bonferroni’s post hoc test.

## Supplementary Material

Supplemental Material

## Data Availability

All data is presented within this manuscript and is available for use upon publication. Campylobacter jejuni induces autoimmune peripheral neuropathy via Sialoadhesin and Interleukin-4 axes datasets are available through the DRYAD databse under https://doi.org/10.5061/dryad.xgxd254hs.

## Highlights

- GBS induced by *C. jejuni* in this mouse model was dependent upon Siglec-1 and IL-4 axes.
- Autoimmunity depended upon IL-4 cytokine production by T helper cells.
- Antiganglioside autoantibodies and nerve lesions were reduced by depletion of IL-4.
- Siglec-1 was essential for colonic T cell maturation and autoantibody elicitation.
- *C. jejuni* sialylated oligosaccharide motifs acted as Siglec-1-ligands for phagocytosis and an epitope for autoimmunity.
- IL-4 or Siglec-1 blockade are suggested as therapeutic interventions against GBS.
