# Supplementary material for: Campylobacter jejuni induces autoimmune peripheral neuropathy via Sialoadhesin and Interleukin-4 axes
Source: Gut Microbes. 2022 Apr 20;14(1):2064706. doi: 10.1080/19490976.2022.2064706 (PMC9037470; doi:10.1080/19490976.2022.2064706)
Supplement: Supplemental Material [file KGMI_A_2064706_SM4065.zip › b_Malik etal_Highlights_REVISED.docx]

**Highlights**

- GBS induced by *C. jejuni* in this mouse model was dependent upon Siglec-1 and IL-4 axes.
- Autoimmunity depended upon IL-4 cytokine production by T helper cells.
- Antiganglioside autoantibodies and nerve lesions were reduced by depletion of IL-4.
- Siglec-1 was essential for colonic T cell maturation and autoantibody elicitation.
- *C. jejuni* sialylated oligosaccharide motifs acted as Siglec-1-ligands for phagocytosis and an epitope for autoimmunity.
- IL-4 or Siglec-1 blockade are suggested as therapeutic interventions against GBS.
