# Supplementary material for: Campylobacter jejuni induces autoimmune peripheral neuropathy via Sialoadhesin and Interleukin-4 axes
Source: Gut Microbes. 2022 Apr 20;14(1):2064706. doi: 10.1080/19490976.2022.2064706 (PMC9037470; doi:10.1080/19490976.2022.2064706)
Supplement: Supplemental Material [file KGMI_A_2064706_SM4065.zip › e_Figure 2_IL4 depletion IHC_compressed.pptx]

## Slide 1
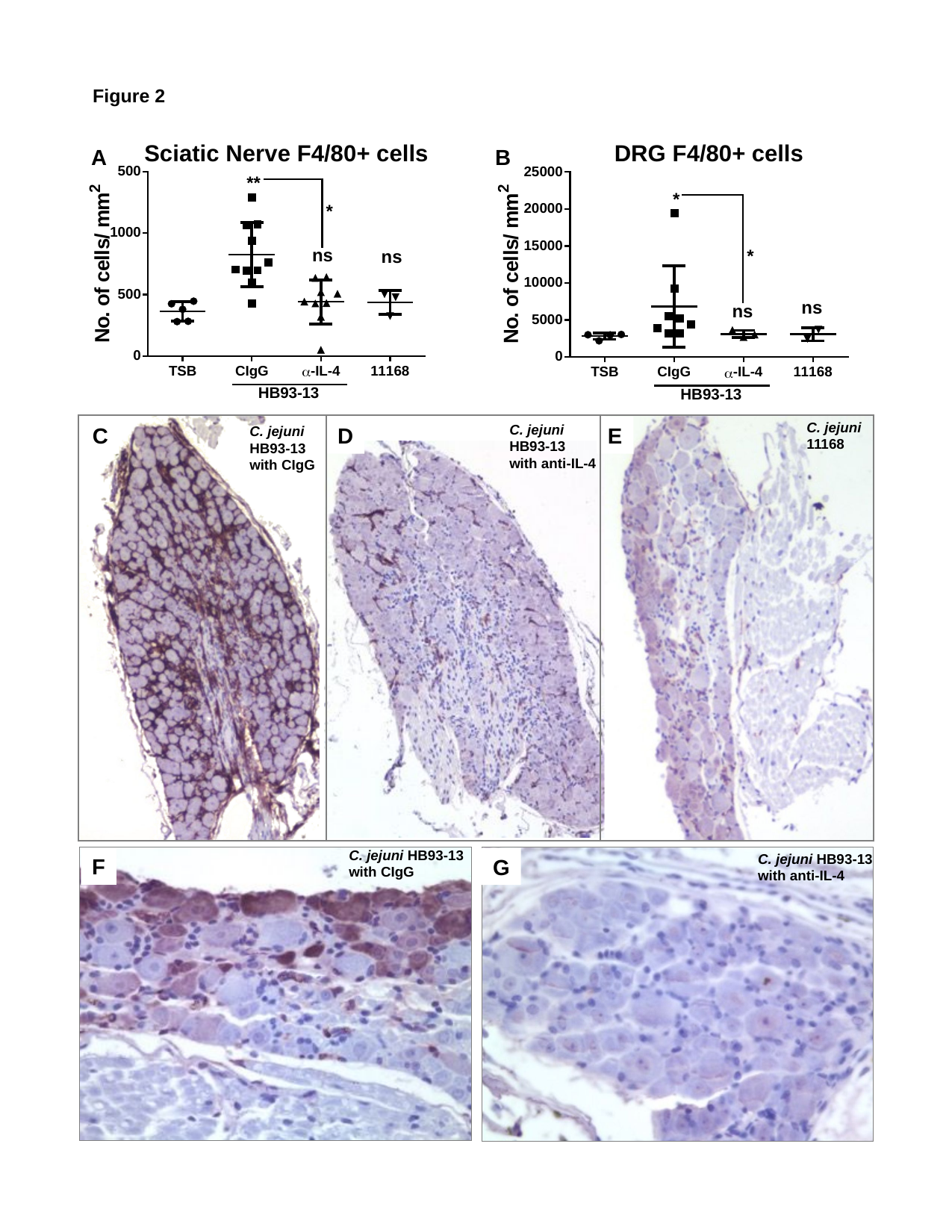

Figure 2
C
D
E
F
G
A
B
C. jejuni 11168
C. jejuni HB93-13
with anti-IL-4
C. jejuni HB93-13
with CIgG
C. jejuni HB93-13
with CIgG
C. jejuni HB93-13
with anti-IL-4
