# Supplementary material for: Campylobacter jejuni induces autoimmune peripheral neuropathy via Sialoadhesin and Interleukin-4 axes
Source: Gut Microbes. 2022 Apr 20;14(1):2064706. doi: 10.1080/19490976.2022.2064706 (PMC9037470; doi:10.1080/19490976.2022.2064706)
Supplement: Supplemental Material [file KGMI_A_2064706_SM4065.zip › j_Figure 7_Mouse experiment data.pptx]

## Slide 1
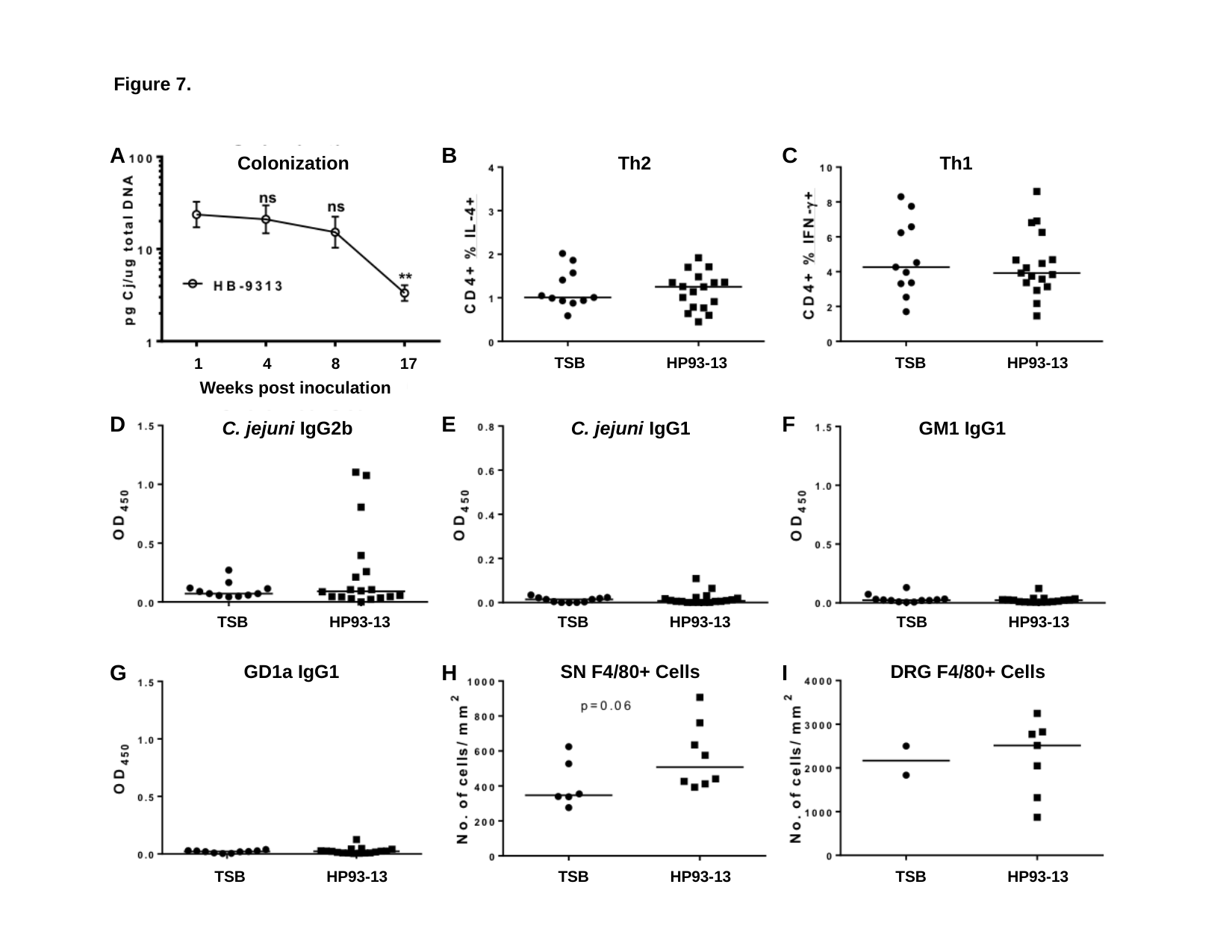

Figure 7.
1 4 8 17
TSB	HP93-13
TSB	HP93-13
Colonization
Th2
Th1
Weeks post inoculation
C. jejuni IgG1
GM1 IgG1
C. jejuni IgG2b
TSB	HP93-13
TSB	HP93-13
TSB	HP93-13
GD1a IgG1
SN F4/80+ Cells
DRG F4/80+ Cells
GD1a IgG1
TSB	HP93-13
TSB	HP93-13
TSB	HP93-13
A
B
C
D
E
F
G
H
I
