# Supplementary material for: Campylobacter jejuni induces autoimmune peripheral neuropathy via Sialoadhesin and Interleukin-4 axes
Source: Gut Microbes. 2022 Apr 20;14(1):2064706. doi: 10.1080/19490976.2022.2064706 (PMC9037470; doi:10.1080/19490976.2022.2064706)
Supplement: Supplemental Material [file KGMI_A_2064706_SM4065.zip › k_Maliketal_Supplementary figures.pptx]

## Slide 1
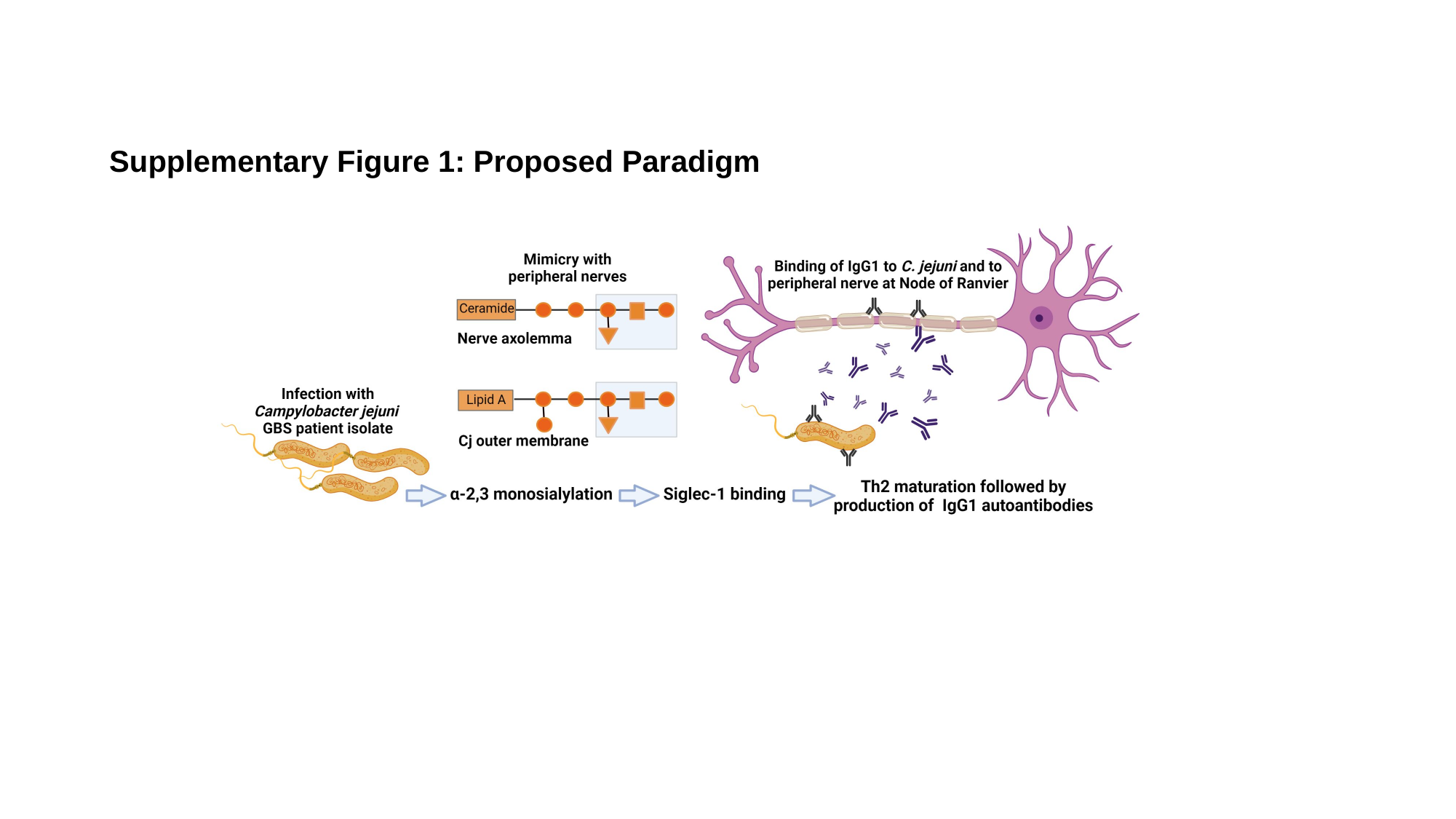

Supplementary Figure 1: Proposed Paradigm

## Slide 2
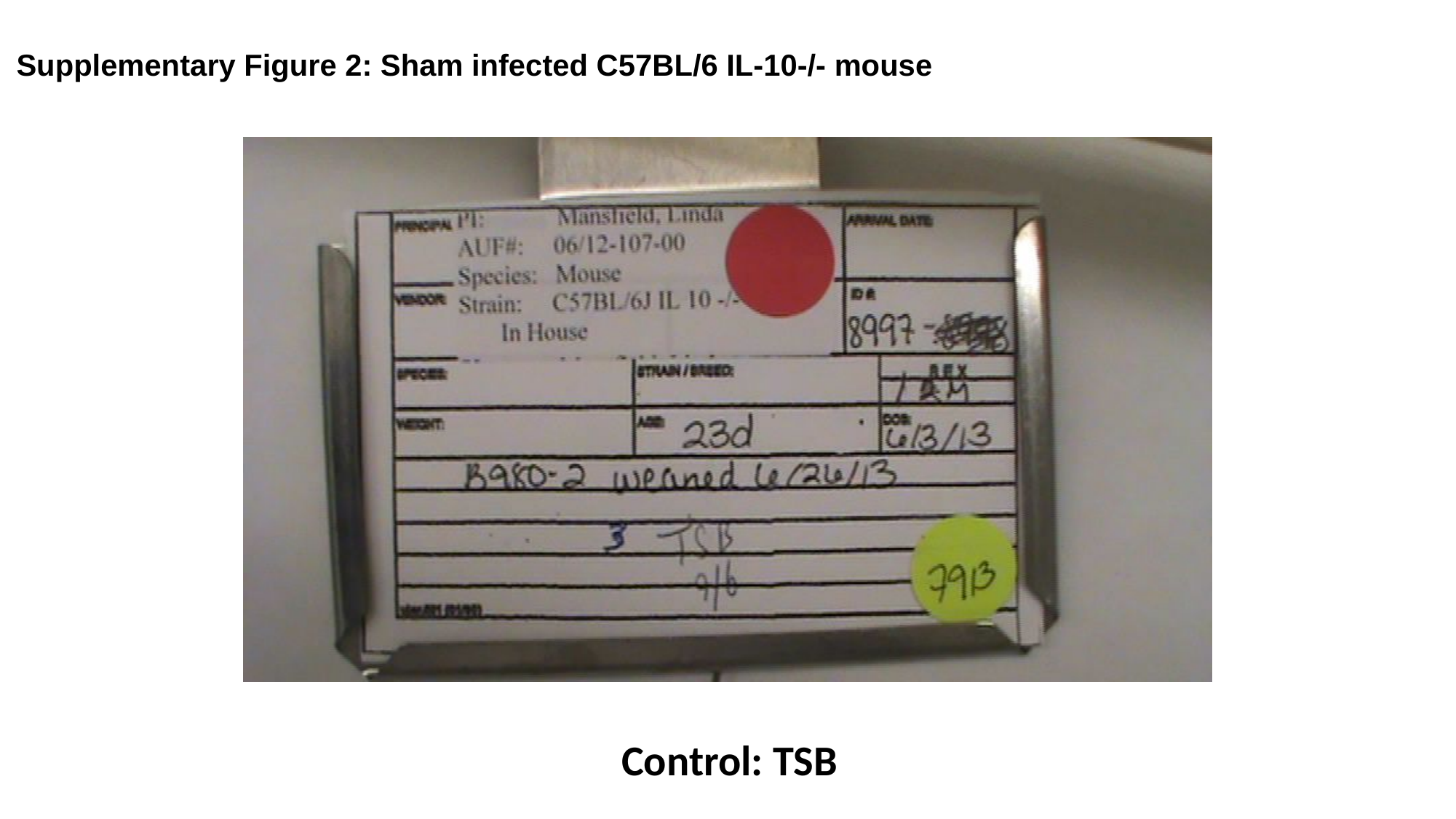

Supplementary Figure 2: Sham infected C57BL/6 IL-10-/- mouse
Control: TSB

## Slide 3
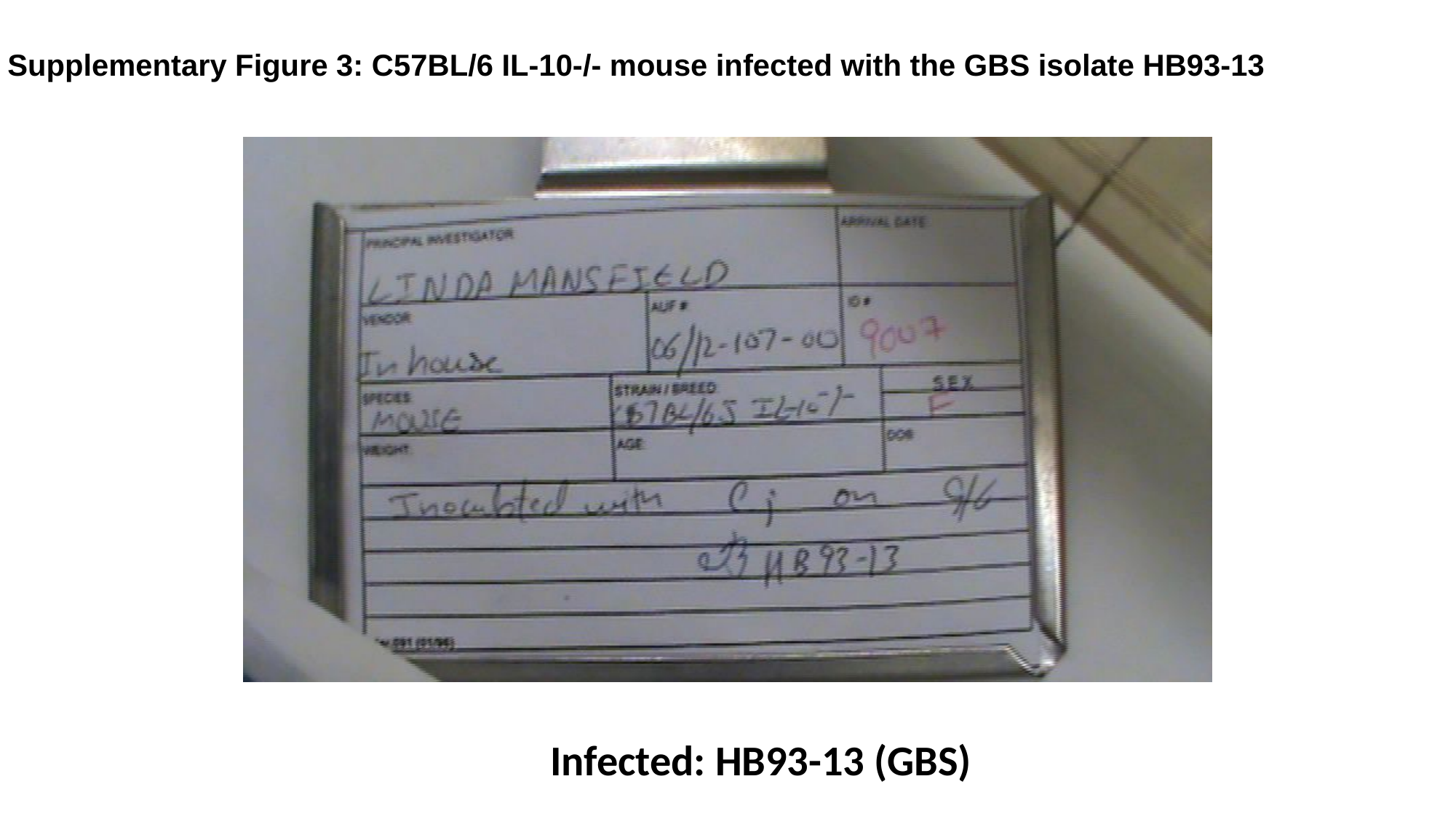

Supplementary Figure 3: C57BL/6 IL-10-/- mouse infected with the GBS isolate HB93-13
Infected: HB93-13 (GBS)
