# Supplementary figures and images for: Campylobacter jejuni induces autoimmune peripheral neuropathy via Sialoadhesin and Interleukin-4 axes
Source: Gut Microbes. 2022 Apr 20;14(1):2064706. doi: 10.1080/19490976.2022.2064706 (PMC9037470; doi:10.1080/19490976.2022.2064706)

## Slide 1
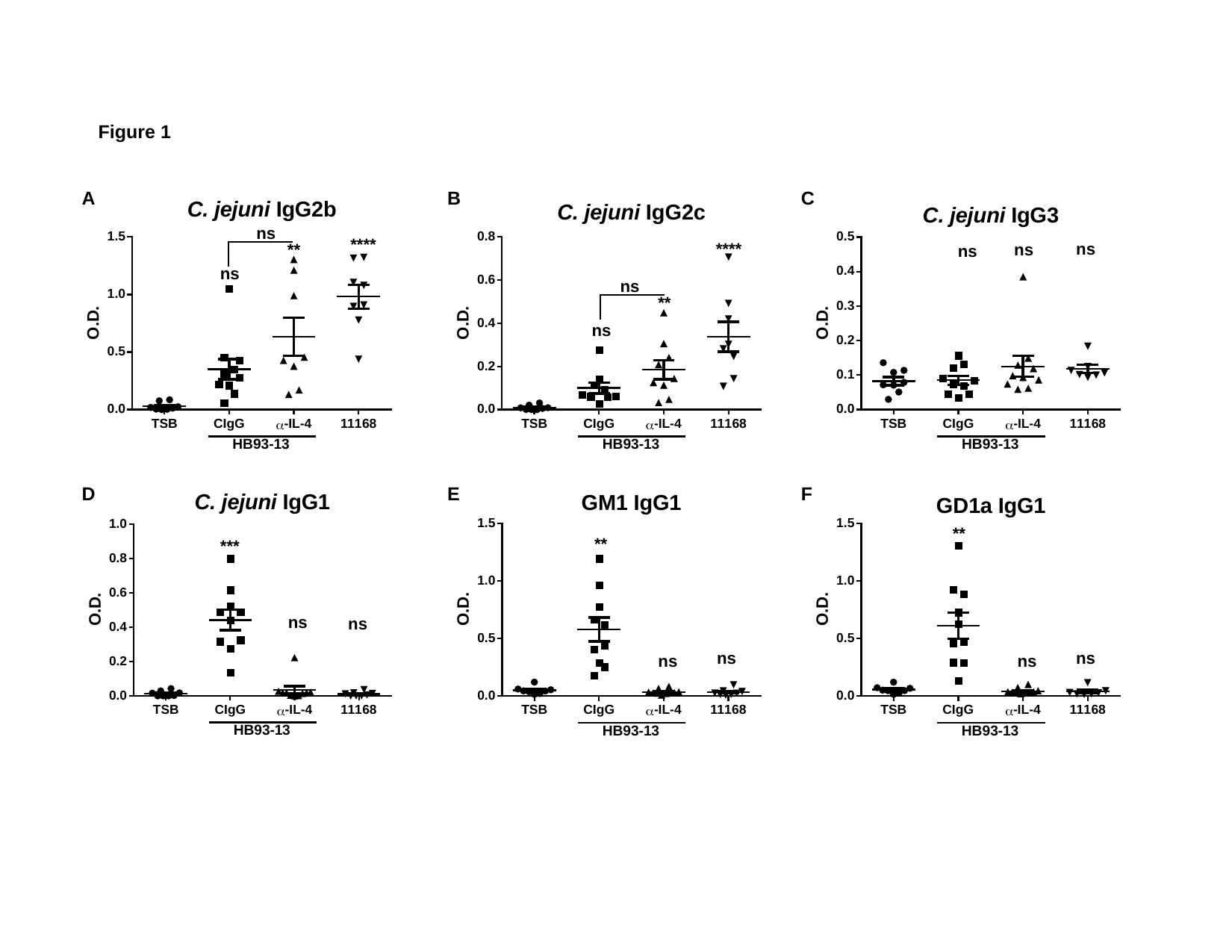

Figure 1
A
B
C
D
E
F

Supplement: Supplemental Material [file KGMI_A_2064706_SM4065.zip › d_Figure 1_Cjejuni and ganglioside IgG.pptx]

## Slide 1
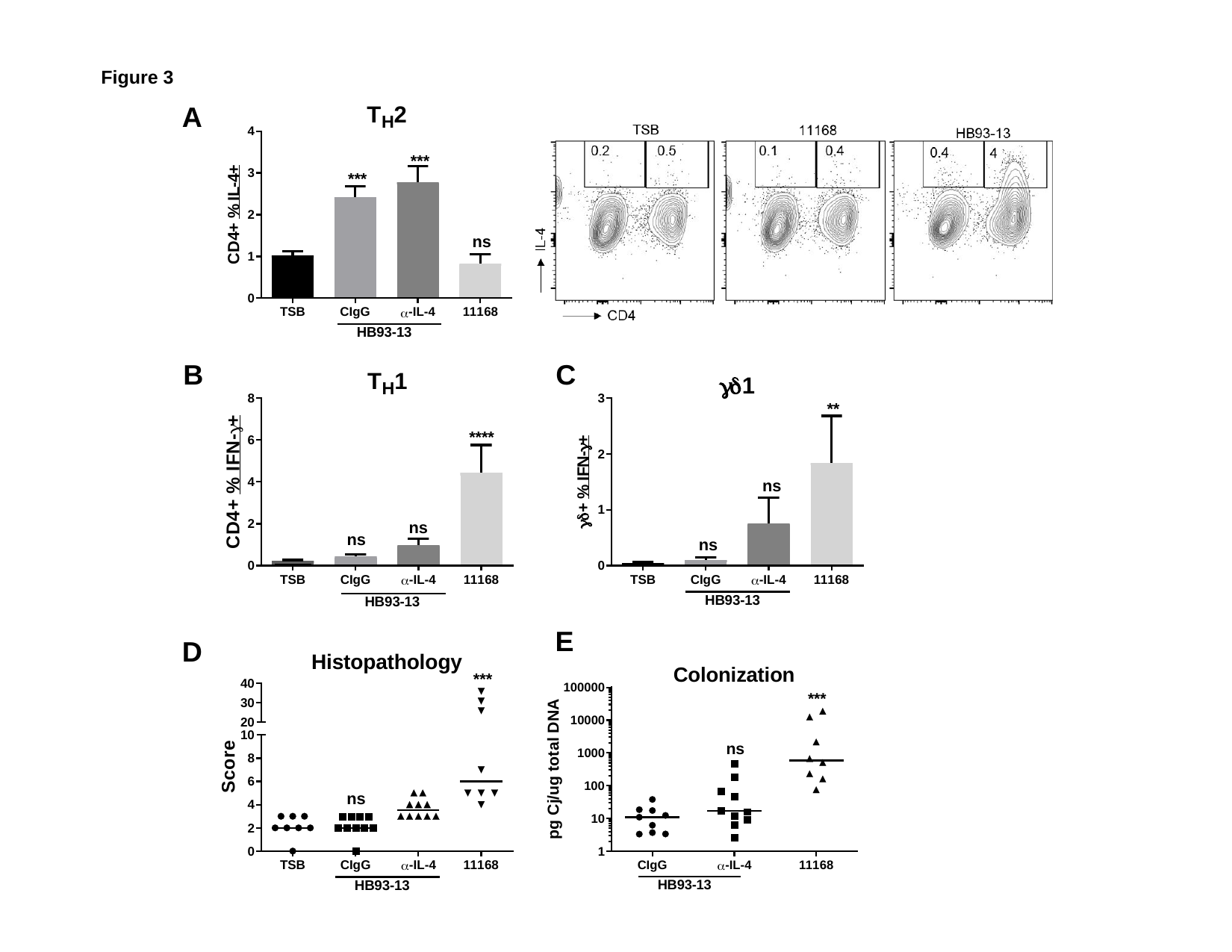

Figure 3
A
B
C
E
D

Supplement: Supplemental Material [file KGMI_A_2064706_SM4065.zip › f_Figure 3_colon measurements.pptx]

## Slide 1
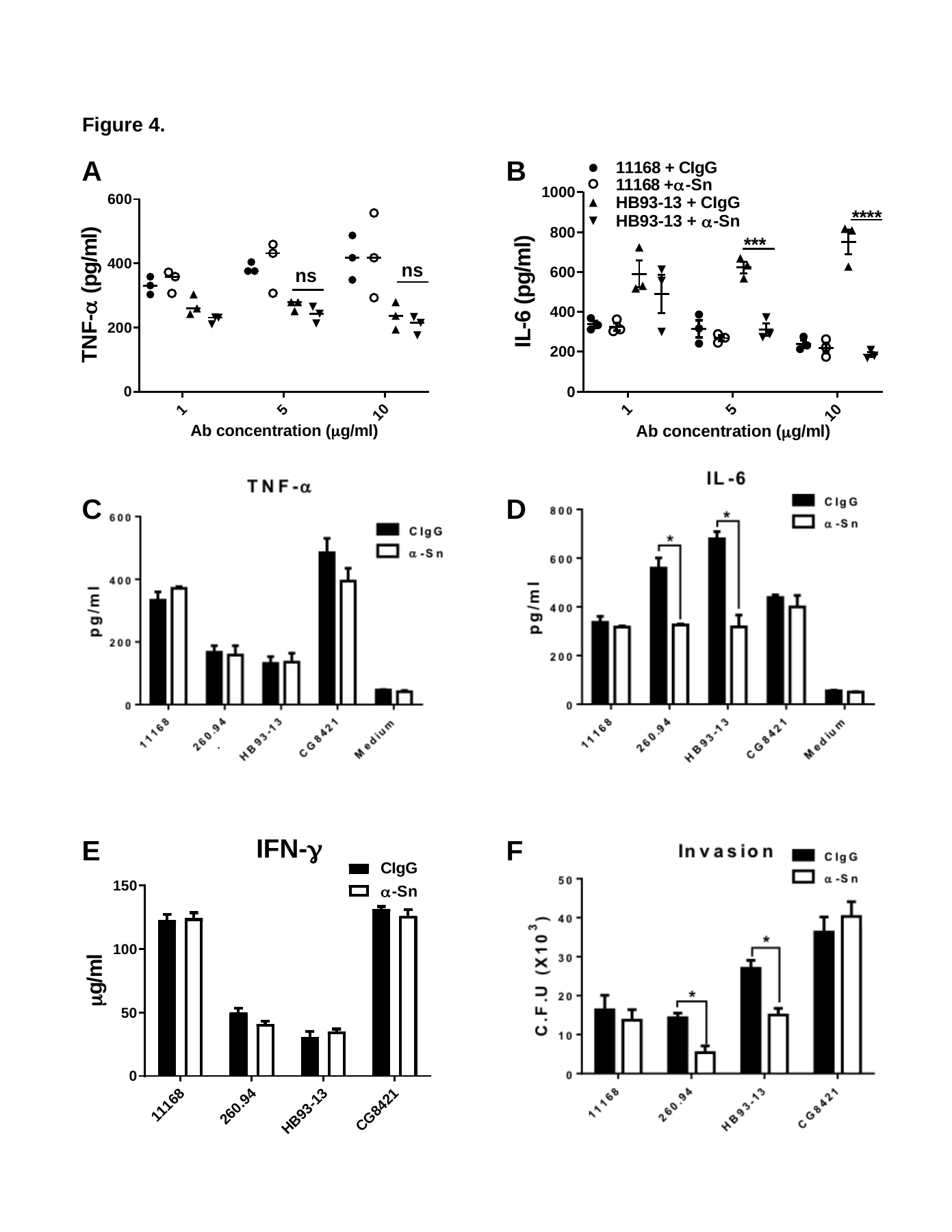

Figure 4.
A
B
C
D
E
F

Supplement: Supplemental Material [file KGMI_A_2064706_SM4065.zip › g_Figure 4_Siglec1 Blocking Exvivo_FINAL.pptx]

## Slide 1
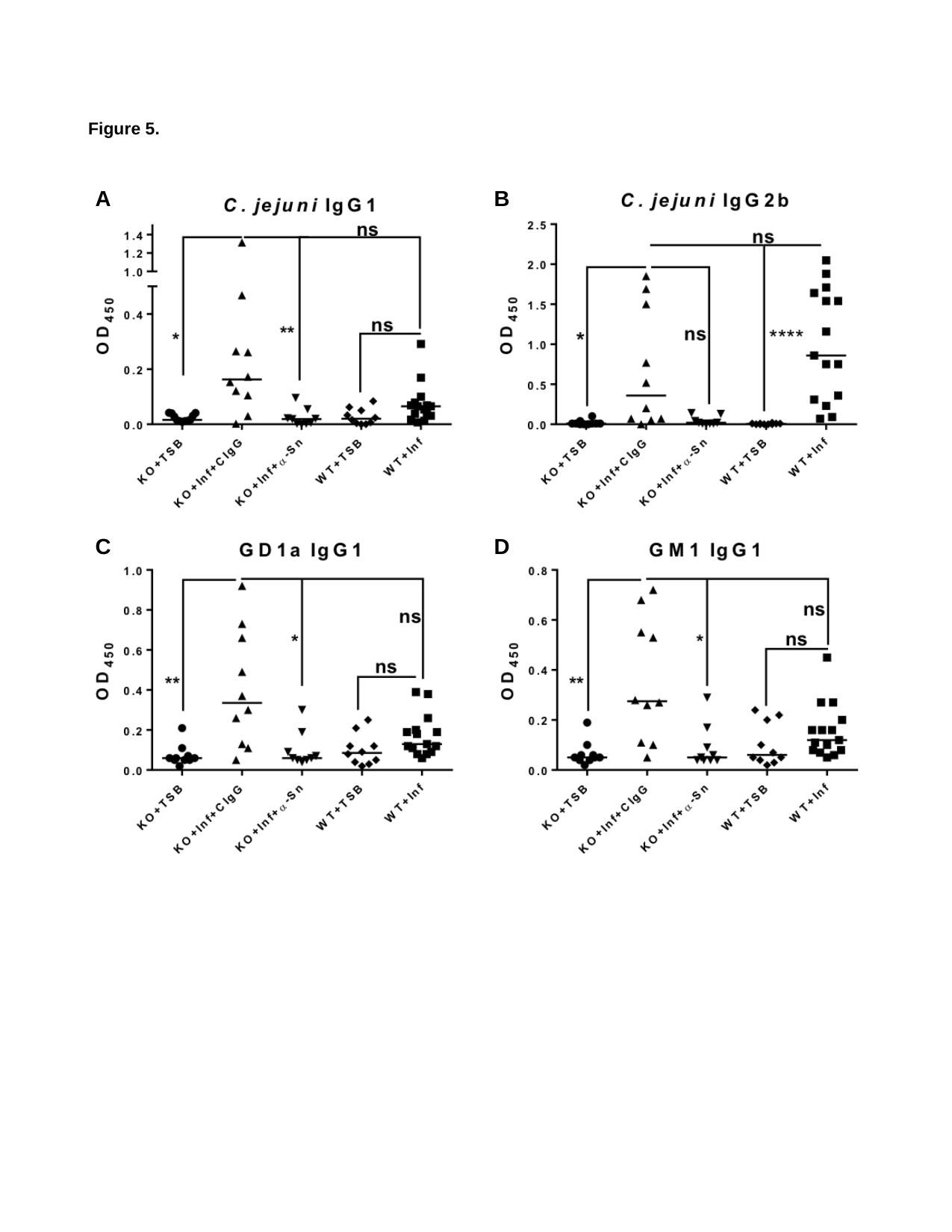

Figure 5.
A
B
C
D

Supplement: Supplemental Material [file KGMI_A_2064706_SM4065.zip › h_Figure 5_Antibody responses with Siglec1 blocking.pptx]

## Slide 1
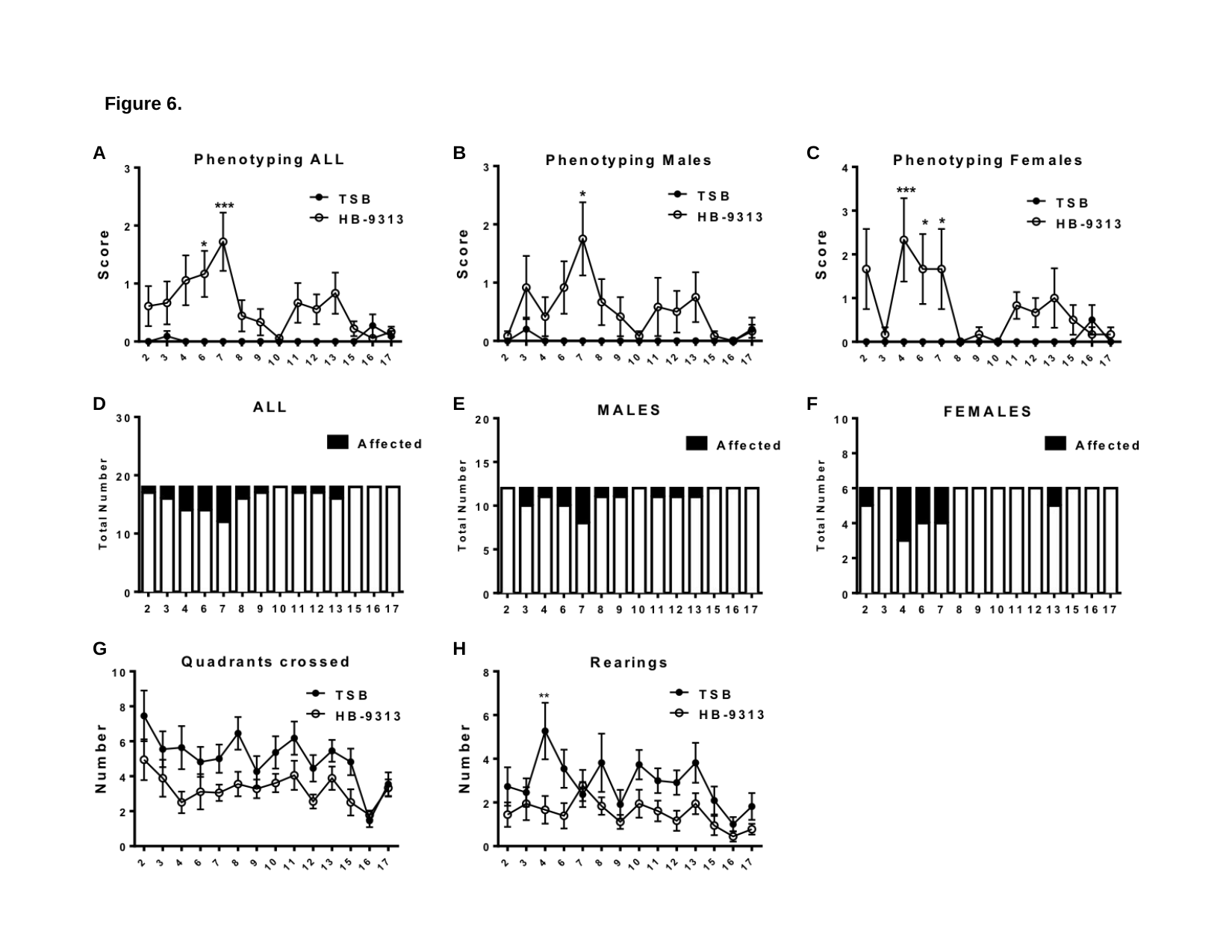

Figure 6.
A
B
C
D
E
F
G
H

Supplement: Supplemental Material [file KGMI_A_2064706_SM4065.zip › i_Figure 6_Neurological Phenotyping_FINAL.pptx]
